# Kinome Analysis of Honeybee (*Apis mellifera L*.) Dark-Eyed Pupae Identifies Biomarkers and Mechanisms of Tolerance to *Varroa* Mite Infestation

**DOI:** 10.1038/s41598-020-58927-2

**Published:** 2020-02-07

**Authors:** Albert J. Robertson, Erin Scruten, Mohammad Mostajeran, Tom Robertson, Connor Denomy, Daniel Hogan, Anna Roesler, Colton Rutherford, Anthony Kusalik, Philip Griebel, Scott Napper

**Affiliations:** 1 Meadow Ridge Enterprises Ltd., Saskatoon, SK Canada; 2 Vaccine and Infectious Disease Organization, University of Saskatchewan, Saskatoon, SK Canada; 3 Department of Biochemistry, Microbiology and Immunology, University of Saskatchewan, Saskatoon, SK Canada; 4 Department of Computer Science, University of Saskatchewan, Saskatoon, SK Canada; 5 School of Public Health, University of Saskatchewan, Saskatoon, SK Canada

**Keywords:** Biochemistry, Biomarkers

## Abstract

The mite *Varroa destructor* is a serious threat to honeybee populations. Selective breeding for *Varroa* mite tolerance could be accelerated by biomarkers within individual bees that could be applied to evaluate a colony phenotype. Previously, we demonstrated differences in kinase-mediated signaling between bees from colonies of extreme phenotypes of mite susceptibility. We expand these findings by defining a panel of 19 phosphorylation events that differ significantly between individual pupae from multiple colonies with distinct *Varroa* mite tolerant phenotypes. The predictive capacity of these biomarkers was evaluated by analyzing uninfested pupae from eight colonies representing a spectrum of mite tolerance. The pool of biomarkers effectively discriminated individual pupae on the basis of colony susceptibility to mite infestation. Kinome analysis of uninfested pupae from mite tolerant colonies highlighted an increased innate immune response capacity. The implication that differences in innate immunity contribute to mite susceptibility is supported by the observation that induction of innate immune signaling responses to infestation is compromised in pupae of the susceptible colonies. Collectively, biomarkers within individual pupae that are predictive of the susceptibility of colonies to mite infestation could provide a molecular tool for selective breeding of tolerant colonies.

## Introduction

Western honeybee (*Apis mellifera L*.) populations are showing increased annual losses worldwide^1–4^. This trend is cause for considerable concern as many food crops depend on honeybees for pollination^5^. A number of causative, or contributing, factors to the declining health of honeybee colonies have been proposed including: *Varroa destructor* (Anderson and Trueman)^6^ parasitism and associated viral pathogens^7,8^, increased use of pesticides, lack of bee genetic diversity, and other factors^9^. Of these, the ectoparasitic mite *Varroa destructor* is typically considered the most significant threat to honeybee health^7,8,10^. In large part this is because these mites serve as a vector for a number of viral pathogens including: Deformed wing virus^7,11^, Israeli acute paralysis virus^12^, Acute bee paralysis virus, and Kashmir bee virus^13^. *Varroa* also compromises bee health by removing hemolymph^14^ and feeding on bees’ fat body tissue^15^.

In response to the threats imposed by *Varroa* mites, many producers have incorporated miticides into their management practices. Unfortunately, these treatments can have detrimental effects on honeybee health and introduce dangerous residues into the wax^16^. Further, as mites develop resistance to these treatments, the use of miticides is unlikely to represent a viable long-term solution^16^. An alternative approach is to focus on selective breeding of honeybees with an enhanced capacity to resist and/or tolerate *Varroa* mite infestation and associated viruses.

The feasibility of genetic selection for *Varroa* mite resistance is supported by the historic example of Asian honeybees (*Apis ceranae*) who, in the face of evolutionary pressures imposed by the parasite, developed protective mechanisms, including behavioral characteristics (such as grooming and hygienic traits) and immune adaptations^17,18^. Western honeybees, whose exposure to this parasite extends only about fifty years, have yet to naturally develop resistance mechanisms and remain susceptible to infestation^17^. Although a number of breeding programs have identified colonies with some resistance to mites, these colonies are lacking in other economic traits, such as honey production^19^. Accelerated breeding of resistance traits within Western honeybees is complicated by our limited understanding of the mechanisms mediating susceptibility to mite parasitism, a shortage of biomarkers to identify resistant progeny, and instability of resistant phenotypes within a colony. Nevertheless, through genetic selection, a number of research teams have established colonies with an increased *Varroa* tolerant phenotype^20–22^. *Varroa* sensitive hygiene (VSH) lines are the best-defined genetic stock able to suppress mite infestation^23,24^. The susceptible and tolerant honeybee colony phenotypes of the current investigation were developed and characterized by the Saskatraz natural selection project (http://www.saskatraz.com) with the goal of increasing the frequency of the alleles associated with economic traits and eventual distribution to commercial beekeepers. This is accomplished through recurrent natural selection of survivor colonies, in the absence of synthetic miticides. The project selects for quantitative traits, including honey production, wintering ability, mite tolerance, and general colony health^25,26^.

A number of molecular approaches, including at the levels of the transcriptome^26–29^ and proteome^30,31^, have been applied to honeybees in efforts to understand the cellular mechanisms of *Varroa* resistance as well as to identify biomarkers of these traits. This is a daunting task due to the potential complexities of the molecular mechanisms underlying these phenotypes, coupled with the challenges of deciphering biology within a mixed genetic population. This last point is particularly true of bees where multiple mating events by individual queen bees, high recombination rates, caste-specific influences on signaling, and supersedure events can increase the variability of individual bees within a colony. In addition, there are inherent limitations to the extent to which patterns of gene and/or protein expression can accurately predict or rationalize complex phenotypes due to a variety of post-transcriptional and post-translation regulatory events.

As protein phosphorylation often serves as a central mechanism for direct regulation of cellular processes, characterization of cellular responses at the level of kinase-mediated phosphorylation (kinome analysis) has the potential to offer unobstructed insight into complex biology^32^. That phosphorylation events can be represented by short peptides, enabling the creation of arrays for high throughput analysis of global cellular kinase activity, in particular in a manner that can be customized for species of interest^33^. Kinome analysis through species-specific peptide arrays has proven to be a powerful approach for deciphering biology, in particular in the context of host-pathogen interactions within outbred populations including cattle^34–36^, pigs^37^, and chickens^38^. Our previous efforts to develop a honeybee-specific kinome array (when applied to bees with a well-defined phenotype) provided evidence for signaling differences between individual bees, representing a variety of developmental stages, selected from colonies representing extreme phenotypes of *Varroa* mite tolerance and susceptibility^25^.

While providing evidence for the potential utility of this technology to identify phosphorylation biomarkers that inform breeding programs, this initial study was limited in that the bees considered represented only a single colony of each phenotype of mite susceptibility. Practical application of the technology would require the capacity to discriminate among bees representing colonies of a spectrum of *Varroa* mite tolerant phenotypes as well as greater consideration of the variability that exists within individuals of the same colony. In our initial kinome investigation, variability among individual kinome profiles was observed, even within bees of the same colony and developmental stage^25^. This variability can reflect the previously discussed unique aspects of bee biology (multiple mating events by individual queen bees, high recombination rates, caste-specific influences on signaling, and supersedure events), individual responses to environmental stimuli, and inherent variability of signaling within individuals’ “kinotypes”^39^. Despite this internal variability, distinct kinome patterns associated with colony phenotypes were observed, motivating further efforts to confirm the effectiveness of the adopted approach.

In the current investigation, we expand on the findings of our previous investigation of the utility for kinome analysis to reveal biomarkers and mechanisms of *Varroa* mite tolerance in honeybees by identifying a panel of 19 phosphorylation events with significant (p < 0.01) differences in phosphorylation when comparing individual pupae (n = 5/phenotype) selected from two colonies of each high and low susceptibility to the parasite. Bees evaluated were at the dark-eyed (white-bodied) pupae stage of development to minimize signaling differences between individuals of the same colony that could result from caste specializations or environmental influences. The utility of these biomarkers was validated by analyzing individual pupae (n = 18) from multiple colonies (n = 8) representing a spectrum of mite susceptibilities. The biomarkers effectively discriminated among individuals on the basis of colony phenotype and survival time. Further, analysis of the kinome profiles indicated that differential susceptibilities to the parasite were associated with innate immune capabilities even in the absence of infestation. Specifically, relative to pupae from *Varroa* mite tolerant colonies, Toll-like receptor (TLR) signaling^40–42^ – a central pathway of the innate immune response – was down-regulated in pupae from mite susceptible colonies. The hypothesis that differential susceptibilities to the parasite were associated with innate immune capabilities is supported by the observation that mite infestation induced elevated innate immune signaling responses in pupae from the tolerant, but not susceptible, colonies. These results indicate that innate immune capabilities contribute to mite resistance, either directly or possibly indirectly by influencing vulnerability to secondary infections by mite-associated viruses. Collectively, in addition to offering insight into the cellular processes underlying *Varroa* mite tolerance, this investigation enabled identification of a group of biomarkers that could be applied at the level of individual pupae to predict phenotypes at a population (colony) level.

## Results

### Characterization of colony phenotypes

Eight colony phenotypes were analyzed in this investigation [Table 1]. Three colonies (S88, S23A, and S14 JHN-13) which showed the longest survival times and maintained lower *Varroa* mite populations over their lifetime were classified as tolerant. Three colonies (S65–15 BC, S88-4, and G4) which showed shorter survival times with mite levels showing sudden dramatic increases in population growth were classified as susceptible. Two colonies (S96-4 JHN-12 and S65 SAT-1) which demonstrated intermediate levels of *Varroa* infestation with shorter survival times than the tolerant colonies, but maintained lower mite levels than the susceptible colonies, were classified as intermediate phenotype [Table 1]. Total honey production of each colony over its survival time, as an indicator of colony strength and productivity, is also presented [Table 1].

**Table 1:** *Varroa* mite Burden and Colony Survival Time.

Phenotype	Tolerant			Intermediate		Susceptible		
Colony ID	S88	S23A	S14 JHN-13	S96-4 JHN-12	S65 SAT-1	S65-15 BC	S88-4	G4
Sampling Date	09-2010	08-2011	07- 2015	08- 2013	09- 2011	08-2016	09-2011	09-2010
Mite Infestation at Sampling Date (MHB)	5.50	0.00	1.69	9.03	16.50	42.42	50.00	67.00
Mean Mite Infestation (MHB)	4.11	5.33	5.26	13.58	5.82	N/A	14.15	52.00
SE ±	1.2	1.4	1.3	9.5	2.2	N/A	7.3	5.2
# Samples	18	26	18	7	10	1	9	4
Total Honey Production (lbs.)	850	568	163	279	509	0	366	0
Colony Survival (Months)	58	48	53	19	24	15	18	17

### Biomarkers of *varroa* mite susceptibility

**Figure 1:**
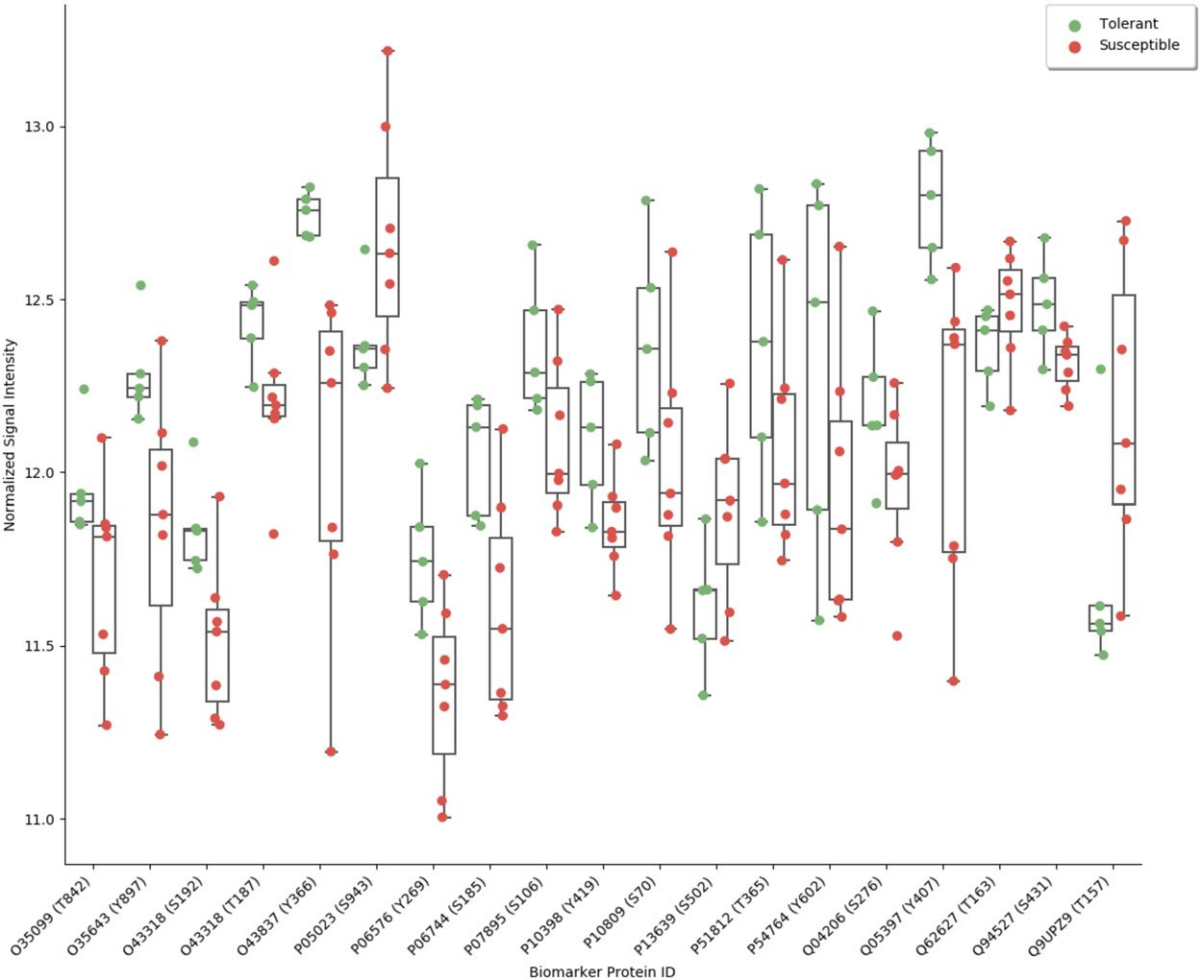
Swarm Plot Illustrating Biomarker Application. For each of the nineteen biomarker peptides, the intensities of signal from pupae from colonies of known phenotype are represented by color-coded dots: *Varroa* mite tolerant (green), *Varroa* mite susceptible (red).

To identify biomarkers associated with *Varroa* mite susceptibility, kinome datasets corresponding to uninfested pupae from colonies representing the extremes of mite susceptibility and tolerance were analyzed. Nineteen peptides had a significant difference (p < 0.01) in levels of phosphorylation when comparing pupae from colonies of the tolerant and susceptible phenotypes [Table 2]. These differentially phosphorylated peptides can be grouped according to biological processes (innate immunity, stress responses, and metabolism) implicated in *Varroa* mite susceptibility^40–42^. With a coefficient of variance of less than one percent, the normalized data for each biomarker peptide was highly consistent across the replicate pupae of the two representative colonies of each phenotype. For many of the biomarker peptides the range of intensities associated with individuals of the susceptible phenotype partially overlap with intensities of phosphorylation of that peptide by individuals of the tolerant phenotype. This likely reflects the complexity of the phenotype as well as diversity of signaling in individual pupae within a colony [Fig. 1].

**Table 2:** Biomarker Peptides: Differently Phosphorylated Peptides Between Pupae Collected from *Varroa* Susceptible and Tolerant Colonies.

	Protein	ID	Sequence	P
Innate Immunity	TAK1 kinase	O43318	YMTNNKGSAAWMAPE	0.001
	TAK1 kinase	O43318	CDLNTYMTNNKGSAA	0.003
	Mitogen-activated protein kinase kinase kinase_5	O35099	TETFTGTLQYMAPE	0.009
	Nuclear factor NF-kappa-B p110 subunit Rel-p110	Q94527	YIQLKRPSDGATSEP	0.005
	Transcription_factor p65 Nuclear factor NF-kappa-B	Q04206	IQLKRPSDGALSEP	0.005
	Focal adhesion kinase 1 FADK1	Q05397	IVDEEGDYSTPATRD	0.005
	AP-1 complex subunit beta-1	O35643	VEGQDMLYQSLKLTN	0.008
Metabolism	ATP synthase_subunit_beta	P06576	TSKVALVYGQMNEPP	0.004
	Na-K transporting ATPase subunit alpha1	P05023	ICKTRRNSLFRQGM	0.009
	Glucose-6-phosphate isomerase	P06744	GPRVHFVSNIDGTHI	0.005
	Isocitrate_dehydrogenase subunit_beta,	O43837	TKDLGGQSSTTEF	0.006
Stress Responses	Ribosomal protein S6 kinase alpha	P51812	DSEFTCKTPKDSPGV	0.006
	Elongation factor 2 (EF-2)	P13639	KVMKFSVSPVVRVAV	0.007
	60_kDa_heat_shock_protein	P10809	ILEQSWGSPKITKDG	0.016
	Superoxide dismutase	P07895	SIFWCNLSPNGG	0.008
Other	Ephrin type-A receptor 4 EPH-like kinase 8 (EK8)	P54764	SYVDPHTYEDPNQAV	0.006
	PRKC_apoptosis_WT1 regulator_protein__	Q62627	LREKRRSTGVVHLPS	0.006
	A-Raf Kinase	P10398	QTAQGMDYLHAKNII	0.010
	Intestinal cell kinase (ICK)	Q9UPZ9	CKIRSRPPYTDYVSTRW	0.010

### Application of kinome biomarkers to individual bees to predict colony phenotype

**Figure 2:**
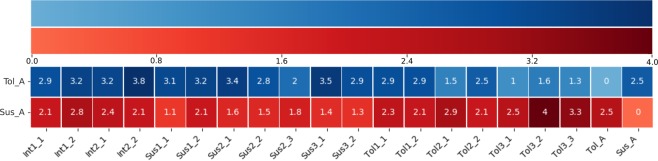
Euclidian Distances of Individual Pupae measured by comparing the phosphorylation levels in each phenotype to the mean phosphorylation levels of the pupae representing the tolerant (Tol_A) and susceptible phenotypes (Sus_A). Higher values represent increasing distance in Euclidian space and decreasing similarity, while low values indicate high similarity and a value of zero represents identical vectors or self-comparison.

The predictive power of the biomarker peptides was evaluated using kinome data from individual, uninfested dark-eyed pupae (n = 18) selected from colonies with a spectrum of susceptibilities to *Varroa* mite infestation. The priority was to determine the effectiveness of biomarkers selected at the level of a colony phenotype in anticipating molecular differences within individual pupae. Kinome analysis was performed blinded to the phenotypes of the individual pupae. From the resulting kinome data, each pupa was assigned scores based on similarity to the mean of the pupae representing the tolerant and susceptible phenotypes based on levels of phosphorylation across the nineteen biomarker peptides [Fig. 2]. From this, a cumulative biomarker susceptibility score was calculated as the difference between the scores relative to the tolerant and susceptible phenotypes [Table 3]. In general, pupae of the same colony had consistent biomarker scores, supporting the feasibility of using this type of approach to guide breeding efforts at the level of a colony.

**Table 3:** Variance in Phosphorylation Levels of Biomarker Peptides Across Individual Pupae of Colonies of Different Mite Susceptibility Phenotypes.

Colony	Pupae	Distance from mean tolerant phenotype^X^	Distance from mean susceptible phenotype^Y^	Biomarker Score (x-y)
G4	Sus2_1	3.4	1.6	1.8
	Sus2_2	2.8	1.5	1.3
	Sus2_3	2	1.8	0.2
S88-4	Sus3_1	3.5	1.4	2.1
	Sus3_2	2.9	1.3	1.6
S65-15 BC	Sus1_1	3.1	1.1	2.0
	Sus1_2	3.2	2.1	1.1
S96-4 JHN-12	Int1_1	2.9	2.1	0.8
	Int1_2	3.2	2.8	0.4
S65 SAT-1	Int2_1	3.2	2.4	0.8
	Int2_2	3.8	2.1	1.7
S14 JHN-13	Tol1_1	2.9	2.3	0.6
	Tol1_2	2.9	2.1	0.8
S23A	Tol2_1	1.5	2.9	−1.4
	Tol2_2	2.5	2.1	−0.4
S88	Tol3_1	1.0	2.5	−1.4
	Tol3_2	1.6	4.0	−2.4
	Tol3_3	1.3	3.3	−2.0

**Figure 3:**
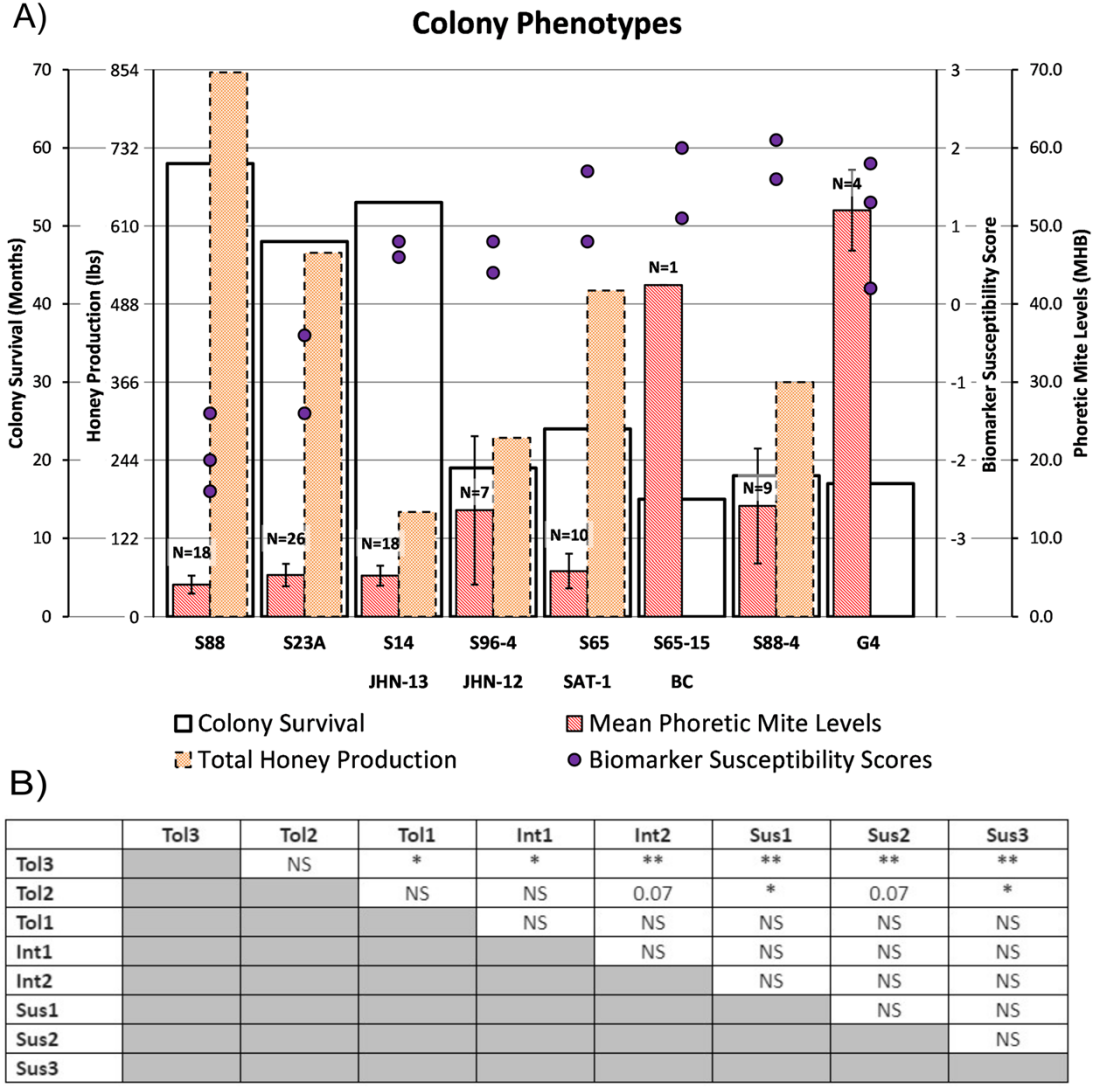
Mite loads, Colony Survival, Honey Production, *Varroa* mite Susceptibility Scores. (**A**) The survival time, mean phoretic mite infestation, honey production, and biomarker susceptibility scores for 8 colony phenotypes are shown here. Error bars are shown as ± SE of the mean phoretic mite level where N is the number of samples tested to calculate the mean [Table 1]; where S65-15 BC is represented only by a single sample. The purple dots represent the biomarker susceptibility scores calculated from the kinome array (n = 299 peptides) analyses of dark eyed pupae. Each dot represents a score calculated from one pupa. (**B**) Significance of difference between biomarker scores 28 possible pairs of colonies. *p < 0.05, **p < 0.01.

When the calculated kinome biomarker scores are compared to the survival times and mite loads of the eight colonies under consideration there is an apparent trend with the observed phenotype, in particular for pupae from colonies of the tolerant phenotype [Fig. 3A]. This trend was confirmed through pairwise analysis of the biomarker scores of the various colonies. In particular, the S88 tolerant colony (Tol3) was significantly different (p < 0.01) from all three susceptible colonies [Fig. 3B]. The biomarker scores of the S88 colony were also significantly different from those of both colonies of intermediate phenotype; p < 0.01 for S96-4 JHN-12 (Int1) and p < 0.05 S65 SAT-1 (Int2). Pupae from the S23A colony (Tol2) had biomarker scores significantly different (p < 0.05) from two of the three susceptible colonies; S65-15BC (Sus1) and S88-4 (Sus3). The biomarker scores of pupae from the tolerant phenotype S14 JHN-13 (Tol1) were not significantly different from those of any of the susceptible colonies but were statistically different (p < 0.05) from those of Tol3 [Fig. 3B]. Collectively indicating that the biomarkers are most effective at distinguishing pupae of the tolerant colonies from those of intermediate and susceptible phenotypes.

### Cellular mechanisms of *varroa* mite susceptibility

To investigate molecular mechanisms of *Varroa* mite tolerance, pathway over-representation analysis was performed on kinome datasets corresponding to uninfested bee pupae (n = 5/phenotype) from two colonies representing each phenotype of Varroa susceptibility^25^. A considerable number of peptides (58 of 299) were differentially phosphorylated (p < 0.05) when comparing pupae representing the two phenotypes. This suggests that these phenotypes reflect complex, multi-faceted molecular mechanisms and that these differences exist even in the absence of *Varroa* mite infestation. Consistent with this hypothesis, a large number of pathways were found to be differentially activated when comparing bees of the two different phenotypes [Table 4]. Notably, relative to bees from the tolerant colonies, there was a down-regulation of Toll-like receptor (TLR) signaling in pupae from susceptible colonies. The process of recurrent natural selection for *Varroa* tolerance may be resulting in the enrichment of genetic mechanisms involved in signaling innate immune responses, in response to pathogens associated with mite infestation. TLR signaling activates antimicrobial factors and other defensive mechanisms at the cellular level^39,40^. This provides for colonies expressing these traits a selective advantage with higher survival rate in presence of mite infestation and largest honey production.

**Table 4:** Pathway Over-Representation Analysis of Uninfested Dark-Eyed Pupae from *Varroa* mite Susceptible versus Tolerant Colonies.

Direction	Pathway	ID	Source	P
Up-Regulated	N-cadherin signaling events	15853	PID NCI	0.01
	beta-catenin independent WNT signaling	17081	REACTOME	0.02
	RAC1 signaling pathway	15344	PID NCI	0.02
	Ca2 + pathway	16886	REACTOME	0.03
	Ctcf: first multivalent nuclear factor	4040	PID BIOCARTA	0.03
	S1P2 pathway	15183	PID NCI	0.03
	TGF-beta receptor signaling	15571	PID NCI	0.04
	ALK1 signaling events	15251	PID NCI	0.04
	Arf1 pathway	15337	PID NCI	0.04
	Glypican 1 network	15119	PID NCI	0.04
	Il12 and stat4 dependent signaling pathway	4054	PID BIOCARTA	0.04
	NRAGE signals death through JNK	13200	REACTOME	0.04
	Regulation of RAC1 activity	14985	PID NCI	0.04
	CDC42 signaling events	15467	PID NCI	0.05
Down-Regulated	ERK1 activation	13000	REACTOME	0.02
	Axon guidance	17789	REACTOME	0.02
	Toll Like Receptor 9 (TLR9) Cascade	13042	REACTOME	0.04
	EPH-ephrin mediated repulsion of cells	19071	REACTOME	0.05

### Global signaling responses to *varroa* mite challenge

**Figure 4:**
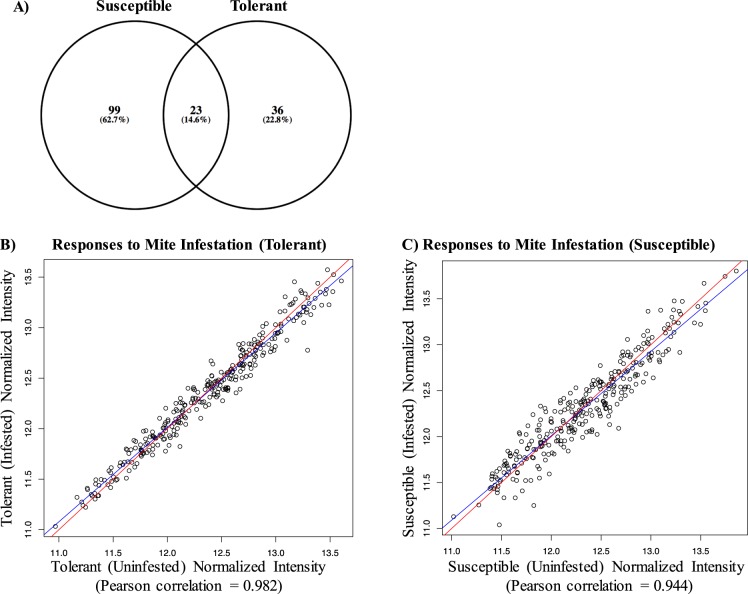
Kinome responses of pupae from colonies susceptible and tolerant to *Varroa* infestation. (**A**) Venn diagram shows the number of differently phosphorylated peptides in susceptible and tolerant pupae in presence of mite infestation. (**B**,**C**) Scatterplots of kinome peptides signal intensity of infested versus uninfested pupae from tolerant and susceptible colonies, respectively.

Kinome responses to *Varroa* mite infestation were evaluated in individual pupae representing susceptible and tolerant phenotypes (n = 5/phenotype) in the presence and absence of mites. In pupae from the tolerant colonies, 59 peptides underwent significant changes (p < 0.05) in their phosphorylation levels in response to mite infestation. In contrast, mite infestation of pupae from susceptible colonies resulted in significant (p < 0.05) changes in the phosphorylation status of 122 peptides, nearly half the array and double the number observed for the pupae from tolerant colonies [Fig. 4A]. Further, the majority of the peptides which underwent mite-induced differential phosphorylation were unique to susceptible pupae and only 23 differentially phosphorylated peptides were common to both phenotypes. Thus, signaling responses induced by the parasite were, to a large extent, distinct when comparing pupae from susceptible and tolerant colonies. This trend for greater kinomic changes within susceptible pupae in response to *Varroa* mite infestation is supported by scatterplots which illustrate a greater number, and magnitude, of changes within pupae of the susceptible phenotype [Fig. 4B,C].

### Pathway analysis of responses to *varroa* mite infestation

To gain insight into the cellular basis of the differential susceptibilities to *Varroa* mite infestation, pathway analysis was performed on the kinome data corresponding to responses to mite infestation in pupae of the tolerant [Table 5] and susceptible [Table 6] phenotypes. Consistent with the observation of a greater number of differentially phosphorylated peptides in susceptible pupae in response to infestation [Fig. 4A], more pathways were differentially activated within pupae from colonies of the susceptible versus the tolerant phenotype. Pupae of the tolerant phenotype displayed activation of pathways relating to innate immune responses, including activation of TLR signaling [Table 5]. In contrast, pupae of the susceptible phenotype displayed no evidence for innate immune activation in response to the parasite. Instead, the upregulated pathways were associated with activation of stress-associated responses [Table 6].

**Table 5:** Pathway Over-Representation Analysis of Responses of Dark-Eyed Pupae from *Varroa* Tolerant Colonies to Mite Infestation.

Direction	Pathway	ID	Source	P
**Tolerant**				
Up-Regulated	TNF receptor signaling pathway	15154	PID NCI	0.01
	Adipocytokine signaling pathway	590	KEGG	0.02
	Protein processing in endoplasmic reticulum	10363	KEGG	0.02
	IL-1 signaling pathway	16110	INOH	0.04
	Toll-like receptor signaling pathway	16121	INOH	0.05
Down- Regulated	None	None	None	None

**Table 6:** Pathway Over-Representation Analysis of Responses of Dark-Eyed Pupae from *Varroa* Susceptible Colonies to Mite Infestation.

Direction	Pathway	ID	Source	P
**Susceptible**				
Up- Regulated	IRE1alpha activates chaperones	13378	REACTOME	9.23E-04
	Unfolded Protein Response (UPR)	16784	REACTOME	9.23E-04
	XBP1(S) activates chaperone genes	13377	REACTOME	0.002
	Metabolism of proteins	18366	REACTOME	0.003
	Dual incision reaction in TC-NER	13847	REACTOME	0.02
	Formation of transcription-coupled NER (TC-NER) repair complex	13844	REACTOME	0.02
	Nucleotide Excision Repair	19742	REACTOME	0.02
	RNA Polymerase I Transcription Initiation	13738	REACTOME	0.02
	Sonic hedgehog receptor ptc1 regulates cell cycle	3999	PID BIOCARTA	0.02
	Transcription-coupled NER (TC-NER)	13848	REACTOME	0.02
	DNA Repair	19821	REACTOME	0.04
	RNA Polymerase I Transcription	16984	REACTOME	0.04
	Regulation of ck1/cdk5 by glutamate receptors	4104	PID BIOCARTA	0.04
	TAK1 mediates p38 MAPK activation	13024	REACTOME	0.04
Down- Regulated	ALK1 signaling events	15251	PID NCI	0.01
	Downregulation of TGF-beta receptor signaling	13267	REACTOME	0.01
	Glypican 1 network	15119	PID NCI	0.01
	Citric acid cycle/respiratory electron transport	17057	REACTOME	0.01
	Signaling by NOTCH1	19096	REACTOME	0.03
	Cytokine-cytokine receptor interaction	515	KEGG	0.03

## Discussion

Previously, through the development and application of a honeybee specific peptide array for kinome analysis, our group provided evidence of differences in kinase-mediated signaling between bees from individual colonies of extreme *Varroa* mite susceptibility phenotypes. These differences were present at a number of developmental stages^25^. These efforts provided insight into the extent, and nature, of the signaling responses within each phenotype to mite infestation^25^. While providing proof-of-principle support for the utility of kinome analysis for identifying phosphorylation-associated biomarkers of mite tolerant phenotypes, the restricted scope of phenotypic diversity considered within this study limited the extent to which these findings can be translated in real-world scenarios. The current work builds on those efforts by investigating pupae representing multiple colonies of each mite susceptibility phenotype to identify biomarkers, as well as to determine the molecular mechanisms, of these traits. Analysis of multiple colonies of each phenotype enables identification of more robust biomarkers and reliable mechanistic explanations of these phenotypes; subsequent evaluation of these biomarkers within colonies of a spectrum of *Varroa* mite susceptibilities represents a more realistic scenario for biomarker application.

A number of investigations have shown honeybees have innate immune responses when challenged with pathogens^25,40–42^. This response could be a contributing factor to *Varroa* mite tolerant phenotypes. Kinome analysis indicates that differences in innate immune capabilities exist between the pupae of tolerant and susceptible phenotypes, both in the presence and absence of infestation. Specifically, that there was a down-regulation of Toll-like receptor (TLR) signaling in pupae from susceptible colonies. As TLR signaling is a central pathway in innate immune responses, the reduced TLR signaling in susceptible bees is consistent with increased susceptibility to mites as well as their associated viral pathogens.

In our previous kinome investigation, difference in innate immunity between the mite susceptible and tolerant phenotypes were only observed in response to mite infestation, which was interpreted as evidence that the molecular mechanism of susceptibility involved mite mediated suppression of innate immune responses rather than differences in innate immune capabilities between the phenotypes in the absence of infestation. In the current investigation, there is still evidence for suppression of innate immune responses in the susceptible bees by *Varroa* mites; however, the differences in innate immunity also appear to precede infestation. Notably, the initial investigation classified the differential responses at the level of gene ontology analysis (which provides a more general overview of biological processes) and considered bees from only a single colony of each phenotype. In contrast, the current investigation utilizes pathway over-representation analysis (which offers more specific insight into biological responses) and considers bees from multiple colonies of each phenotype, which collectively should offer more reliable and detailed insight into the biological differences between the phenotypes.

A panel of nineteen peptides showing different phosphorylation levels between uninfested pupae collected from mite susceptible and tolerant colonies were identified. The biological roles of the phosphorylation events represented by these biomarker peptides implicated differences in innate immunity, metabolism, and stress tolerance between the two phenotypes. For many of the individual biomarker peptides there is incomplete separation of pupae based on phenotype, which likely reflects the complexity of the phenotype as well as diversity of signaling in individual pupae. However, as a collective unit, these peptides are effective in predicting colony phenotype, in particular of identifying pupae of tolerant colonies. This emphasized the necessity to consider multiple peptides to effectively discriminate between tolerant and susceptible pupae. As would be expected, the biomarker peptides were less effective in discriminating colonies with corresponding reduction in colony survival time from intermediate phenotypes.

In the current investigation, emphasis was placed on the identification of biomarkers that discriminate individuals from colonies of the extreme phenotypes. This reflects the priority of bee breeding efforts in selecting for, or against, colonies with mite tolerance or susceptibility. From a practical perspective, identifying pupae representing either tolerant or susceptible phenotypes would be an effective approach for guiding breeding efforts. Importantly, these potential biomarkers were detected in the absence of mite infestation, indicating that these differences reflect innate (naturally selected) traits rather than differences in responses to infestation. The colonies that show these innate traits were previously subjected to natural selection in the presence of *Varroa* without miticide treatments (www.saskatraz.com). For bee breeding efforts, it would be important to have selectable markers that do not depend on infestation. Having established those biomarkers, future investigations will consider greater representation of colonies of intermediate phenotype to more definitively establish the accuracy of this approach.

The biomarker scores were generally consistent for pupae of the same colony, supporting the feasibility of using this type of approach to guide breeding efforts at the level of a colony. A degree of biological variability of individual pupae of a given colony is anticipated due to multiple mating events of individual queen bees, high recombination rates, and supersedure events. From a practical perspective, this would require that multiple pupae from a particular colony are analyzed to make a reliable determination of colony phenotype. Future investigations with greater numbers of pupae from individual colonies should be completed to determine the minimum number of individual pupae, and specific stage of development, which provides the most reliable prediction of colony phenotype. Such efforts will need to pay particular consideration to the priorities of application and the consequences associated with false positives versus false negatives. In the interest of developing a cost-effective screening tool, such investigations may also consider whether it is possible to obtain reliable colony phenotype predictions by performing kinome analysis on a pooled sample of 30 or more pupae from an individual colony.

Collectively, while the current investigation provides strong proof-of-principle support for the utility of kinome analysis for identifying phosphorylation-associated biomarker of the *Varroa* mite tolerance phenotype, further investigations which consider a greater number of bees from each colony, as well as a greater number of phenotypically distinct colonies, will ultimately determine the accuracy, and value, of these biomarkers. Alternatively, kinome analysis could be used to identify biological events which, from cost and practicality perspectives, are more amenable for use by bee breeders. For example, a previous kinome investigation of stress responses of livestock identified differences in signaling associated with carbohydrate metabolisms between high and low stress responding animals. This led to the demonstration that serum glucose levels, which are easy and inexpensive to monitor, had a greater predictive value of stress-associated behaviors than traditional stress biomarkers^43,44^.

In conclusion, the process of recurrent natural selection is resulting in phenotypes that can survive longer in the presence of *Varroa* infestations. This is due to a number of behavioural traits such as grooming and hygienic behaviour (VSH). However, in this work we have also shown some evidence that tolerant colonies are showing increased innate immune capabilities that would increase the ability of these colonies to tolerate the pathogens associated with *Varroa* infestation. In future kinome analyses it would be beneficial to look at other stocks of honeybees showing *Varroa* resistance for evidence of innate immune responses.

## Materials and Methods

### Colony phenotype selection

A detailed description of the honeybee breeding and selection program used to construct and identify the *Varroa* mite susceptible and tolerant phenotypes can be accessed at http://www.saskatraz.com and in *Robertson*, *2014*^25^. The Saskatraz natural selection apiaries were initially established with a diverse group of colonies represented by selections from a number of different races. Canadian (*carnica* and *ligustica*), Russian (*caucasica*, *ligustica*, *carnica*), and German (*carnica*) as well as hybrids such as Buckfast. The Saskatraz bees result from natural selection of a mixture of these races selected for honey production, winter survival, and mite tolerance in Saskatchewan, CA (52.1332° N, 106.6700° W). The natural selection apiaries were isolated for close population mating and ranged in size 32–40 colonies.

In this investigation, we studied eight colonies classified as either tolerant, susceptible, or intermediate phenotypes with respect to *Varroa* mite burdens and colony survival times [Table 1**;** Fig. 2]. This represented three colonies of tolerant (S88, S23A, and S14 JHN-13) and susceptible (S65-15 BC, S88-4, and G4) phenotypes, as well as two colonies deemed as an intermediate (S96-4 JHN-12 and S65 SAT-1) phenotype. Colonies were selected between 2010 and 2016. The length of survival under natural selection conditions defines the susceptibility or tolerance to *Varroa* infestation. S65-15 BC was identified as being very susceptible to mite population growth during 2016 with a survival time of 15 months [Table 1].

Data was collected periodically on the phoretic mite infestation from April – September/October each year at each of our natural selection apiaries. *Varroa* infestations on adult bees (phoretic phase) were determined by washing 200–300 bees from each colony in 100% methanol. Mite infestation levels are reported as mites per hundred bees (MHB). MHB = (# of Mites/# of Bees) * 100. For all natural selection apiaries honey production was measured by weighing all supers of honey harvested from each colony over the production season for each year that the colony was alive. Colonies with the defined phenotypes of susceptible, intermediate, and tolerant were subjected to brood analyses. Sealed brood frames were removed from the colonies and several stages of pupae (white, pink, dark-eyed, and dark-bodied) were randomly sampled from each frame from each colony in the month described [Table 1]. Clean stainless-steel forceps were used to open each brood cell and the pupae were removed and visually inspected for the presence or absence of *Varroa*. The pupae were immediately placed in liquid nitrogen after removal and stored at −80 °C until analysis. *Varroa* free pupae were defined as those that did not have a foundress, progeny, or any evidence of *Varroa* in the brood cell (i.e. *Varroa* feces).

All colonies except S65-15 BC were maintained in natural selection apiaries, where no miticide treatments were used. S65-15 BC was observed to be extremely susceptible to *Varroa* population growth in one of our commercial apiaries in August 2016. It was managed for honey production and treated for mites with Apistan (2 strips) on Sep 1, 2015 and Apivar (2 strips) and oxalic (3.2 grams per 100 ml in 50% w/v sucrose) on Apr 18, 2016. None of the other colonies in this apiary showed notable *Varroa* infestations. It is difficult to detect Varroa susceptible colonies because they die quickly.

### Honey production

Honey production is measured by weighing all honey harvested from each colony over the honey production season for each year that the colony was alive.

## Kinome Analysis

### Peptide arrays for kinome analysis

The kinome peptide array customized for the honeybee phosphoproteome has been described^23^. The current investigation utilized the same array with no further modifications. Kinome analyses were performed on 36 individual dark-eyed pupae (18 mite-infested; 18 uninfested) collected from eight different colonies representing a phenotypic range of susceptibility to *Varroa* mite infestation. Prioritizing bees at the dark-eyed pupae stage of development minimizes potential signaling variability arising from different castes or environmental influences in adult bees. All samples were analyzed within the same kinome assay to minimize technical effects due to inter-assay variability. Individual frozen pupae were placed in a sealed plastic bag in the presence of 300 μl of lysis buffer^25^. The pupae were pulverized with a rubber mallet and the resulting suspension was centrifuged at 10,000 × g for 10 min. Supernatants were used for kinome analysis^25^.

### Data analysis

The dataset for each array contains signal intensities associated with the nine technical replicates for each of the 299 peptides spotted on each array. Kinome data were processed through PIIKA 2, a pipeline for processing kinome array data^45^.

### Identification of peptide biomarkers of *varroa* mite susceptibility

A one-sided paired student’s *t*-test of normal distribution was used to compare normalized signal intensity values for each of the 299 peptides of individual pupae (n = 5) representing the two high and low colony phenotypes of *Varroa* mite susceptibility. Specifically, mite tolerance was represented by pupae from the S88 (n = 3) and S23A (n = 2) colonies while mite susceptibility was represented by pupae from the G4 (n = 3) and S88-4 (n = 2) colonies in the absence of mite infestation. Peptides with significant (*P*-value < 0.01) differences in levels of phosphorylation between pupae of the two phenotypes were classified as potential biomarkers.

### Application of kinome biomarkers to individual bees to predict colony phenotype

The predictive power of the biomarker peptides was evaluated using kinome data from individual, uninfested dark-eyed pupae (n = 18) selected from colonies with a spectrum of susceptibilities to *Varroa* mite infestation. This consideration included the pupae from the colonies used to identify the biomarker peptides as well as additional pupae from colonies representing a range of phenotypes, including those classified as tolerant (S88 (n = 3), S23A (n = 2), and S14 JHN13 (n = 2)), susceptible (G4 (n = 3), S88-4 (n = 2), and S65-15 BC (n = 2)) and intermediate (S96-4 JHN12 (n = 2) and S65 SAT-1 (n = 2)) phenotypes all in the absence of *Varroa* mite infestation. Each pupa was assigned scores based on similarity to the mean of the pupae representing the tolerant and susceptible phenotypes based on levels of phosphorylation across the nineteen biomarker peptides. The similarity between phenotypes’ phosphorylation pattern was assessed using pairwise distance from python package scipy (scipy.distance.spatial.pdist). For each phenotype, a vector was created consisting of the normalized values of 19 peptides. The Euclidian distance between each vector and a vector consisting of the mean of the same 19 peptides values in the most tolerant phenotypes (S88 and S23A) was measured, and the same was done with the most susceptible phenotypes (S88-4 and G4). Differences between the biomarker scores of the eight different colonies were analyzed using one-way ANOVA for multiple comparisons using the R package emmeans. Pairwise comparisons were assessed using the post-hoc Tukey HSD test.

### Treatment-treatment variability analysis

For each peptide, a paired *t*-test was used to compare its normalized signal intensity values in the presence and absence of mite infestation. Peptides with significant (*P*-value < 0.10) changes in phosphorylation were identified. This level of significance was chosen to retain as much data as possible to facilitate subsequent pathway analysis^35^.

### Pathway analysis

Pathway analysis was performed as described previously using the software InnateDB utilizing a hypergeometric algorithm with the Benjamini Hochberg correction method^46^.
